# The Youth Anxiety Measure for DSM-5 (YAM-5): Development and First Psychometric Evidence of a New Scale for Assessing Anxiety Disorders Symptoms of Children and Adolescents

**DOI:** 10.1007/s10578-016-0648-1

**Published:** 2016-05-14

**Authors:** Peter Muris, Ellin Simon, Hester Lijphart, Arjan Bos, William Hale, Kelly Schmeitz, International Child and Adolescent Anxiety Assessment Expert Group (ICAAAEG), Anne Marie Albano, Yair Bar-Haim, Katja Beesdo-Baum, Deborah Beidel, Patrick Bender, Jessica Borelli, Suzanne Broeren, Sam Cartwright-Hatton, Michelle Craske, Erika Crawford, Cathy Creswell, Diogo DeSousa, Helen Dodd, Thalia Eley, Barbara Hoff Esbjørn, Jennifer Hudson, Eva de Hullu, Lara Farrell, Andy Field, Lorraine Fliek, Luis Joaquin Garcia-Lopez, Amie Grills, Julie Hadwin, Sanne Hogendoorn, Lindsay Holly, Jorg Huijding, Shin-ichi Ishikawa, Philip Kendall, Susanne Knappe, Richard LeBeau, Einar Leikanger, Kathryn Lester, Helene Loxton, Lauren McLellan, Cor Meesters, Maaike Nauta, Thomas Ollendick, Ana Pereira, Armando Pina, Ron Rapee, Avi Sadeh, Susan Spence, Eric A. Storch, Leonie Vreeke, Polly Waite, Lidewij Wolters

**Affiliations:** 1 Clinical Psychological Science, Faculty of Psychology and Neuroscience, Maastricht University, P.O. Box 616, 6200 MD Maastricht, The Netherlands; 2 Stellenbosch University, Stellenbosch, South Africa; 3 Virenze-RIAGG Maastricht, Maastricht, The Netherlands; 4 Open University, Heerlen, The Netherlands; 5 Utrecht University, Utrecht, The Netherlands

**Keywords:** Youth Anxiety Measure for DSM-5, Questionnaire, Anxiety disorders symptoms, Children and adolescents

## Abstract

The Youth Anxiety Measure for DSM-5 (YAM-5) is a new self- and parent-report questionnaire to assess anxiety disorder symptoms in children and adolescents in terms of the contemporary classification system. International panels of childhood anxiety researchers and clinicians were used to construct a scale consisting of two parts: part one consists of 28 items and measures the major anxiety disorders including separation anxiety disorder, selective mutism, social anxiety disorder, panic disorder, and generalized anxiety disorder, whereas part two contains 22 items that focus on specific phobias and (given its overlap with situational phobias) agoraphobia. In general, the face validity of the new scale was good; most of its items were successfully linked to the intended anxiety disorders. Notable exceptions were the selective mutism items, which were frequently considered as symptoms of social anxiety disorder, and some specific phobia items especially of the natural environment, situational and other type, that were regularly assigned to an incorrect category. A preliminary investigation of the YAM-5 in non-clinical (*N* = 132) and clinically referred (*N* = 64) children and adolescents indicated that the measure was easy to complete by youngsters. In addition, support was found for the psychometric qualities of the measure: that is, the internal consistency was good for both parts, as well as for most of the subscales, the parent–child agreement appeared satisfactory, and there was also evidence for the validity of the scale. The YAM-5 holds promise as a tool for assessing anxiety disorder symptoms in children and adolescents.

## Introduction

Anxiety disorders are among the most prevalent psychiatric problems in children and adolescents. On the basis of a large-scale, longitudinal, epidemiological study, it was concluded that almost 1 in 10 (i.e., 9.9 %) of the young people suffer from one or more anxiety disorders before the age of 16 [1]. Anxiety disorders cause significant impairment in youths’ emotional, social, and academic functioning [2], and typically follow a chronic course, even into adulthood [3], while increasing the risk for other types of psychopathology, in particular depression [4]. Given this, research on childhood anxiety disorders is important, and a considerable number of studies have focused on factors involved in the origins of these problems and their effective treatment. On the basis of a review of studies conducted between 1982 and 2006, Muris and Broeren [5] concluded that “the research on childhood anxiety disorders has made significant advancements” (p. 388), and inspection of the more recent literature indicates that this scientific progress has continued during the last decade.

Self-report questionnaires are widely employed for measuring the frequency and intensity of anxiety symptoms in children and adolescents. This type of assessment is easy to administer, requires a minimum amount of time, and captures information about anxiety symptoms from the child’s point of view [6]. The latter is important because anxiety disorders belong to the category of internalizing (emotional) problems, which are often less observable than the externalizing (behavioral) problems (such as oppositional-defiant disorder and conduct disorder), even to people in the young person’s direct environment. The measurement of anxiety by means of questionnaires is particularly useful for researchers who want to study variations in children’s and adolescents’ anxiety symptoms in relation to hypothesized vulnerability, risk, and protective factors in order to learn more about the mechanisms underlying this psychopathology. Further, within the context of treatment outcome studies on childhood anxiety disorders, such measures are needed in order to quantify the effectiveness of treatment [7]. Besides these purposes, self-report scales are useful in clinical practice or in community/school settings where they can be efficiently employed to detect fear and anxiety in youths, measure the severity of these complaints, and evaluate therapeutic progress [8].

Three of the most commonly used scales for assessing symptoms of fear and anxiety in children and adolescents are the State-Trait Anxiety Inventory for Children [9], the Revised Children’s Manifest Anxiety Scale [10], and the Fear Survey Schedule for Children-Revised [11]. Although support has been found for the reliability and validity of each of these measures, a clear shortcoming is that they are not directly related to the anxiety disorders as listed in the commonly employed Diagnostic and Statistical Manual of Mental Disorders (DSM). This results in a gap between the outcome of the anxiety assessment and the anxiety disorder classification, which hinders communication about youths’ anxiety problems for both clinicians and researchers. Moreover, there is evidence that childhood anxiety symptoms cluster into categories that are consistent with the anxiety disorders in the DSM [12], providing empirical justification for a DSM-based assessment.

After the introduction of the DSM-IV [13], several questionnaires were developed that measure anxiety symptoms in children and adolescents in terms of the DSM nosology. The Multidimensional Anxiety Scale for Children [14], the Screen for Child Anxiety Related Emotional Disorders (SCARED) [15], and the Spence Children’s Anxiety Scale [16] are psychometrically sound examples of such measures. All these scales assess children’s fear and anxiety symptoms in relation to stimuli and situations that are related to diagnostic categories including separation anxiety disorder, social anxiety disorder, generalized anxiety disorder, and panic disorder, although only the revised version of the SCARED (SCARED-R) [17, 18] measures symptoms of the full spectrum of anxiety disorders that according to DSM-IV may occur in youth. Psychometric evaluations of these questionnaires have generally provided positive evidence for their reliability and validity [19], and there are even indications that the new scales are superior in some regards (e.g., sensitivity to measure treatment effects) as compared to the more traditional childhood anxiety questionnaires [20].

With the publication of DSM-5 [21] various changes in the classification of anxiety disorders have been instituted, which may have implications for their assessment. First, obsessive–compulsive disorder and posttraumatic or acute stress disorder are no longer considered as pure anxiety disorders [22, 23] and have been moved to different sections in the DSM, and thus these symptoms no longer need to be captured by childhood anxiety questionnaires. Second, agoraphobia is now regarded as distinct from panic disorder [24], and as such may require additional items in order to strengthen the assessment of this anxiety problem. Finally, given increasing evidence that anxiety is a prominent feature of selective mutism [25], this type of childhood psychopathology is now conceptualized as an anxiety disorder and so standardized assessment should aim to assess for this presentation.

The Anxiety Disorders subgroup of the DSM-5 Anxiety, Obsessive–Compulsive Spectrum, Posttraumatic, and Dissociative Disorders workgroup developed dimensional scales to assess symptoms present in children and adolescents with anxiety disorders [26]. These dimensional scales employ a similar format for each anxiety disorder: first a definition is provided of the disorder, after which 10 uniform questions are asked that assess the frequency of cognitive, physiological, and behavioral symptoms associated with that specific anxiety disorder. A first study exploring the psychometric properties of the DSM-5 dimensional anxiety scales in 8- to 13-year-old children [27] yielded some positive results for their reliability and validity. However, convergent and discriminant validity of the dimensional scales (as investigated through correlations with corresponding and non-corresponding scales on another self-report anxiety scale, the SCARED) were less satisfactory, and this also appeared true for the parent–child and father–mother agreement indices. Besides these psychometric imperfections, the DSM-5 dimensional scales do not include selective mutism and only assess symptoms in relation to one type of specific phobia (the one chosen by the child/adolescent or parent as being most prominent). To deal with this drawback, the instrument could be easily expanded. However, by adding 10 items for each extra anxiety disorder this would result in a fairly large instrument. Further, it is possible that the procedure of asking respondents to repeatedly complete the very same 10 questions for each anxiety disorder could cause fatigue or might have unintended carry-over effects that compromise the quality of the anxiety assessment. For example, respondents might lose interest leading to a careless completion of the measure, or may choose to answer similar questions in the same way in order to make a consistent impression.

In view of these limitations of the dimensional anxiety scales, there remains a need for a stimulus/situation-based questionnaire that assesses anxiety disorder symptoms of children and adolescents in terms of domains that correspond with the classifications that are currently described in the DSM-5. This article describes the development of such a scale, which included the following steps. First, items were created reflecting symptoms of separation anxiety disorder, selective mutism, social anxiety disorder, generalized anxiety disorder, panic disorder, agoraphobia and various types of specific phobias. Then, two waves of expert validity checks, one carried out by an international panel of childhood anxiety researchers and one conducted by clinicians working with anxious children and adolescents, led to the construction of a final version of the new questionnaire, the Youth Anxiety Measure for DSM-5 (YAM-5). The YAM-5 was developed to measure anxiety symptoms in clinical and non-clinical children and adolescents aged 8–18 years (not only as a self-report but also from the perspective of their parents). The scale incorporates all anxiety disorders that are listed in DSM-5, including the ‘new’ category of selective mutism and devotes more attention to the separate entity of agoraphobia and various types of specific phobias. Finally, the YAM-5 was completed by non-clinical youths (12–17 years) and clinically referred children and adolescents (8–18 years). This provided an impression of the suitability of the YAM-5 for young people in this age range. In addition, the reliability (internal consistency) was examined, and, because the parent version as well as a number of other measures were administered in the clinical sample, it was also possible to investigate the parent–child agreement and various aspects of validity.

## Method

### Face Validity Checks

An initial pool of 74 items was created, which was then sent by email to 50 research experts on childhood anxiety disorders. Forty-four experts (i.e., 88 %) responded positively and became members of the International Child and Adolescent Anxiety Assessment Expert Group (ICAAAEG), a panel of psychologists and psychiatrists from the United States of America (*n* = 10), the Netherlands (*n* = 9), the United Kingdom (*n* = 8), Australia (*n* = 5) and various other countries (Brazil, Denmark, Germany, Israel, Japan, Norway, Portugal, South-Africa, and Spain). The experts in the Netherlands were given the Dutch version of this initial questionnaire, while experts from other countries received the English version, which was obtained following a forward- and back-translation procedure which was carried out by a native English speaker and an official translator. All experts were asked to perform a face validity check on the 74 items, which were presented to them in random order. They were asked to indicate for each item which anxiety disorder(s) it probably represented (choosing from the following 10 DSM-5 based categories: separation anxiety disorder, selective mutism, specific phobia—animal type, specific phobia—natural environment type, specific phobia—blood–injection–injury type, specific phobia—situational type/agoraphobia, specific phobia—other, social anxiety disorder, panic disorder, and generalized anxiety disorder), and to comment on the quality of the wording of the item (this could pertain to the specific content, wording, readability, and age-appropriateness of the item). Following this procedure, items were retained, modified (in this case the official translator was involved again), or removed, which resulted in a final version of the questionnaire, the Youth Anxiety Measure for DSM-5 (YAM-5), which consisted of two parts: major anxiety disorders and specific phobias/agoraphobia (see below). The new scale was then given to an international panel of 34 clinicians from Australia, the Netherlands, Portugal, the United Kingdom, and the United States of America, who all worked with children and adolescents with anxiety disorders, for a second face validity check. The clinicians were instructed to indicate only the most probable anxiety disorder for each item (choosing from five categories in the case of major anxiety disorders: separation anxiety disorder, selective mutism, social anxiety disorder, panic disorder, and generalized anxiety disorder, and from five categories in the case of specific phobias/agoraphobia: specific phobia—animal type, specific phobia—natural environment type, specific phobia—blood–injection–injury type, specific phobia—situational type/agoraphobia, and specific phobia—other).

### Suitability and Psychometric Properties of the YAM-5

To investigate the suitability and some psychometric properties of the YAM-5, data were collected in two separate samples. The first sample consisted of 132 non-clinical adolescents (56 boys and 76 girls) aged between 12 and 17 years (*M* = 14.8 years, *SD* = 1.09), who were randomly recruited from a regular high school in the Southern part of the Netherlands. They completed the new questionnaire (in Dutch) group-wise during regular classes as a part of a survey examining the relationship between self-related constructs and psychopathology in youth [28]. Most of the young participants were from original Dutch descent (i.e., >90 %), and all of them had a good mastery of the Dutch language. Participants from three educational levels were included: 16.7 % followed low- or middle-level preparatory vocational education, 34.1 % higher general continued education, and 49.2 % pre-university secondary education. Based on the occupations of both parents, it was estimated that 20.5 % of the participants had a low, 58.3 % a middle, and 21.2 % a high socio-economic background. Before participation, written informed consent was obtained from the child and parents (80 % of those who were approached for this study decided to participate). The study was officially approved by the Ethical Committee of Psychology (ECP) at Maastricht University.

The second sample was composed of 64 children and adolescents (24 boys and 40 girls) aged 8–18 years (*M* = 12.4 years, *SD* = 3.1; 8- to 12-year-olds: *n* = 31, 13- to 18-year-olds: *n* = 33) and their parents, who were recruited at the child and adolescent division of the Maastricht Community Mental Health Center (Virenze-RIAGG) in Maastricht, the Netherlands. Youths completed the YAM-5 individually as part of the regular intake assessment. Nearly all parents of the children and adolescents *(n* = 63) completed the parent-version of the YAM-5 questionnaire. In most cases the parent-version of the YAM-5 was completed by the mother (74 %); in other cases the scale was completed by the father, both parents, or another caregiver (e.g., foster parent). The vast majority of the families was from original Dutch descent (i.e., >95 %) and based on the educational levels of the parents, 14.4 % had a low, 47.6 % a middle, and 38 % a high socio-economic status.

Within the clinical sample, 21 children and adolescents had a primary diagnosis of an anxiety disorder (*M* age = 12.8, *SD* = 3.0, range 8-18 years, 9 boys and 12 girls). Most of them were classified with an anxiety disorder not otherwise specified (*n* = 15); others had generalized anxiety disorder (*n* = 2), social phobia (*n* = 1), specific phobia (*n* = 1), or a disorder in which anxiety played a prominent role (i.e., obsessive–compulsive disorder: *n* = 1, and posttraumatic stress disorder: *n* = 1). It should be noted that a substantial proportion of the children in this clinical anxiety disorders group (76.2 %) also had a comorbid diagnosis, with disruptive behavior disorders (*n* = 7) and mood disorders (*n* = 5) being the most frequent. The remaining 43 children and adolescents in the clinical sample (*M* age = 12.3, *SD* = 3.2, range 8-18 years, 15 boys and 28 girls) were not diagnosed with an anxiety disorder and thus formed the clinical control group; these youths received a variety of diagnoses among which autism spectrum disorder, attention-deficit/hyperactivity disorder, and other disruptive behavior disorders were most common.

Besides the YAM-5 (child and parent report), a number of other instruments were administered during the intake at the mental health center. First, the Junior SCID is the DSM-5-based adaptation of the Kid SCID [29], a semi-structured interview performed with parent and child to generate the most common psychiatric diagnoses in childhood. In the present study, we primarily focused on the anxiety disorders section, and because the sample size was too small to analyze the separate anxiety categories, a total score was derived by summing the anxiety symptoms that were rated as being present in the child, which could be correlated with YAM-5 to evaluate its concurrent validity. Second, the Achenbach scales [30] are widely employed for assessing mental health problems in youth. In this study, the forms to be completed by parents (the Child Behavior Checklist) and children themselves—from 11 years onwards—(the Youth Self-Report) were taken to compute scores of internalizing and externalizing. The former includes emotional problems such as fear and anxiety and thus was employed to investigate the convergent validity of the YAM-5, whereas the latter incorporates behavioral problems and thus was used to examine the divergent validity of the scale.

## Results

### Initial Face Validity Check by Research Experts

Table 1 presents the initial set of 74 items ordered in terms of the anxiety disorders they were intended to measure. The table also shows the percentage of the research experts confirming the intended anxiety disorder classification (i.e., sensitivity), as well as the percentage of the experts indicating alternative classifications (i.e., specificity). Below we discuss the results for each anxiety disorder and also clarify the decision process of maintaining, changing, or removing items, which eventually led to the construction of the final version of the new questionnaire. Maximizing sensitivity and specificity was the leading principle that guided the decision to maintain or eliminate items, while also striving for a parsimonious set of items that formed a good representation of the main characteristics of various anxiety disorders.

**Table 1:** Results of the face validity check of the initial pool of 74 YAM-5 items as performed by the international panel of research experts (*N* = 44) on childhood anxiety disorders

Anxiety disorder item	Confirmation by research experts (%) (sensitivity)	Alternative anxiety disorder (%)^a^ (specificity)	Decision (reason)
*Separation anxiety disorder*			
I am afraid to go anywhere without my parents	100	4.5	Retained: YAM-5-I item 1
I get frightened if my parents leave the house without me	100	6.8	Retained: YAM-5-I item 6
I am afraid that my parents will leave and never come back	100	0	Retained: YAM-5-I item 10
I am afraid that something bad will happen, so I’ll never see my parents again	100	4.5	Retained: YAM-5-I item 15
I am afraid if I am not at home	68.2	59.1 (SITAGO)	Removed (ambiguous item)
I want my father and mother to be with me when I go to sleep	100	13.6	Removed (no explicit anxiety)
I only want to sleep over at another kid’s home if my parents come	100	2.3	Removed (unlikely scenario)
I have very scary dreams that I lose my parents	100	0	Retained: YAM-5-I item 19
I don’t feel well when I have to go somewhere without my parents	100	0	Retained: YAM-5-I item 24
	**96.5**	**9.6**	
*Selective mutism*			
At school I don’t dare to talk to the teacher	86.4	59.1 (SOC)	Retained but changed: YAM-5-I item 2
If I meet someone I don’t know well, I don’t dare to say anything	77.3	68.2 (SOC)	Retained but changed: YAM-5-I item 11
If I come across someone who wants to talk to me, I don’t say anything back	93.2	36.4 (SOC)	Removed (redundant item)
At school I don’t dare to talk to the kids in my class	86.4	59.1 (SOC)	Retained but changed: YAM-5-I item 20
If there is a new visitor at our home, I won’t say anything	95.5	50.0 (SOC)	Retained but changed: YAM-5-I item 25
I am so afraid or shy that I don’t speak at all	95.5	22.7 (SOC)	Removed (partly measures temperament)
In the past I did not dare to say anything at school	93.2	38.7 (SOC)	Removed (measures past symptom)
In the past I did not dare to talk to strangers	84.1	59.1 (SOC)	Removed (measures past symptom)
	**89.0**	**49.2**	
*Social anxiety disorder*			
I find it scary to be with people I don’t know well	97.7	13.6	Retained but changed: YAM-5-I item 3
I find it very scary to talk with people I don’t know	95.5	36.4 (SM)	Removed (ambiguous/redundant item)
I find it scary to eat or drink if other people are looking at me	100	0	Retained: YAM-5-I item 7
I am afraid of being bullied at school	84.1	54.5 (GAD)	Removed (ambiguous item)
I find it very scary to act in a play	100	15.9 (SM)	Removed (not applicable to all children)
I am afraid that I will blush	100	9.1	Retained but changed: YAM-5-I item 12
I am afraid I’ll do something embarrassing	100	0	Retained: YAM-5-I item 16
I am very afraid that other kids don’t like me	100	22.7 (GAD)	Retained: YAM-5-I item 23
I am afraid that other people can see that I’m nervous	95.5	13.7 (PAN)	Removed (ambiguous item)
I am afraid I can’t get the words out	77.3	52.3 (SM)	Removed (ambiguous item)
I find it scary to give a speech in front of the class	100	13.6 (SM)	Retained but changed: YAM-5-I item 28
	**95.5**	**21.1**	
*Panic disorder*			
I panic for no reason	100	4.6	Retained: YAM-5-I item 4
I suffer from panic attacks	100	9.1	Retained but changed: YAM-5-I item 8
If I am afraid my heart beats very quickly	97.7	47.7 (All)	Retained but changed: YAM-5-I item 13
If I am afraid I sweat a lot	95.5	61.4 (All, SOC)	Removed (ambiguous item)
If I am scared I afraid to die	95.5	18.2	Retained but changed: YAM-5-I item 17
If I am afraid I shake a lot	95.5	50.0 (All)	Retained but changed: YAM-5-I item 21
If I am afraid I feel dizzy	100	31.8 (All)	Removed (less common symptom)
I am afraid of having a new anxiety or panic attack	97.7	13.6	Retained: YAM-5-I item 26
I am afraid that other people can see when I am panicking	68.2	63.6 (SOC)	Removed (ambiguous item)
In a big store I am afraid I will panic	65.9	70.5 (SITAGO)	Removed (ambiguous item)
	**91.6**	**37.1**	
*Generalized anxiety disorder*			
I worry about a lot of things	100	0	Retained: YAM-5-I item 5
I worry a lot	100	4.6	Removed (redundant item)
I think a lot about what can go wrong	100	11.4	Retained: YAM-5-I item 9
I worry about everything	100	0	Removed (redundant item)
I find it hard to stop worrying	100	0	Retained: YAM-5-I item 14
I worry a lot about how well I do things	90.9	25.0	Removed (ambiguous item)
I worry a lot about not doing well at school	93.2	13.7 (SOC)	Retained: YAM-5-I item 18
I worry a lot about disasters (for example earthquake, flood)	47.7	70.5 (NATENV)	Retained but changed: YAM-5-I item 22
I worry a lot about wars	90.9	11.4	Retained but changed: YAM-5-I item 22
I don’t feel well because I worry so much	100	0	Retained: YAM-I item 27
	**92.3**	**13.7**	
*Specific phobia—animal type*			
I am afraid of wasps	100	0	Retained: YAM-5-II item 1
I am afraid of dogs	100	0	Retained: YAM-5-II item 3
I am afraid of spiders	100	0	Retained: YAM-5-II item 9
I am afraid of snakes	100	0	Retained: YAM-5-II item 13
I am afraid of cats	100	0	Retained: YAM-5-II item 18
	**100**	**0**	
*Specific phobia—natural environment type*			
I am afraid of the dark	65.9	45.5 (SITAGO, OTH)	Retained: YAM-5-II item 4
I am afraid of standing on a high place	90.9	13.6 (SITAGO)	Retained but changed: YAM-II item 6
I am afraid of thunderstorms	93.2	6.8	Retained: YAM-5-II item 10
I am afraid to swim in deep water	97.7	2.3	Retained: YAM-5-II item 12
	**86.9**	**17.1**	
*Specific phobia—blood–injection–injury type*			
I am afraid of getting an injection	100	0	Retained: YAM-5-II item 11
I am afraid of getting a physical examination in the hospital	81.8	29.5	Retained but changed: YAM-II item 15
I am afraid of blood	100	0	Retained: YAM-5-II item 19
	**93.9**	**7.4**	
*Specific phobia—situational type/Agoraphobia*			
I am afraid to travel in an airplane	84.1	25.0 (OTH)	Retained: YAM-5-II item 5
I am afraid when crossing a large town square	90.9	11.4	Retained: YAM-5-II item 7
I am afraid of places with a lot of people	84.1	50.0 (SOC)	Retained but changed: YAM-5-II item 16
I am afraid when travelling by bus or train	97.7	13.6	Retained: YAM-5-II item 17
I am afraid when travelling by car	88.6	38.6 (GAD)	Removed (ambiguous/redundant item)
I am afraid to cross a long bridge	81.8	29.5 (NATENV)	Removed (ambiguous item)
I am afraid when sailing on a boat	77.3	38.6 (NATENV)	Removed (ambiguous item)
I am afraid to go in an elevator	93.2	15.9	Retained: YAM-5-II item 21
I am afraid to go outside on my own	68.2	68.2 (SEP)	Removed (ambiguous item)
I am afraid to go through a long tunnel	93.2	9.1	Retained: YAM-5-II item 22
	**85.9**	**30.0**	
*Specific phobia—other type*			
I am afraid of loud noises	77.3	25.0 (NATENV)	Retained: YAM-5-II item 2
I am afraid of people who are dressed up in costumes	95.5	6.8	Retained: YAM-5-II item 8
I am afraid that I have to vomit	70.5	38.6 (PAN/BII)	Retained but changed: YAM-5-II item 14
I am afraid that I will choke	50.0	54.5 (PAN)	Retained but changed: YAM-5-II item 20
	**73.3**	**31.2**	

YAM-5 = Youth Anxiety Measure for DSM-5, YAM-5-I = YAM-5 Section I. Major anxiety disorders, YAM-5-II = YAM-5 Section II. Phobias

*SITAGO* specific phobia—situational type/agoraphobia, *SOC* social anxiety disorder, *SM* selective mutism, *GAD* generalized anxiety disorder, *PAN* panic disorder, *All* relevant for all anxiety disorders, *NATENV* specific phobia—natural environment type, *OTH* specific phobia—other, *SEP* separation anxiety disorder, *BII* specific phobia—blood–injection–injury type

^a^Alternative anxiety disorder(s) is (are) only specified if indicated by more than 10 % of the experts. Experts were allowed to indicate more than one disorder for each item, so percentages add up to more than 100 %. Average percentages per anxiety disorder are presented in bold

#### Separation Anxiety Disorder

The majority of items measuring this type of anxiety were satisfactory in terms of sensitivity and specificity. The only exception was the item “I am afraid if I am not at home”: 31.8 % of the experts did not think of the classification of separation anxiety disorder, whereas 59.1 % indicated an alternative classification, most often agoraphobia, which guided our decision to remove this item. In addition, using the qualitative input of the experts, we decided to eliminate two further items (for specific reasons, see Table 1), leaving six items to be retained for the final scale.

#### Selective Mutism

The items that were developed to measure selective mutism were reasonably sensitive given that, on average, 89.0 % of the experts linked these items to this new anxiety disorder. Specificity of items was rather low: 49.2 % of the experts indicated that these items also reflected symptoms of social anxiety disorder. Although this is in line with studies showing considerable overlap between selective mutism and social anxiety disorder [25, 31], we decided to follow the suggestion made by a number of experts to focus items only on the key symptom of failure to speak and to remove any references to anxiety or fear as a motive for this behavior. In addition, four items were deleted because they were either considered as redundant, partly measured temperament, or assessed children’s mute behavior in the past (these were initially included to assess the developmental aspect of this problem, but eliminated because the YAM-5 purports to measure current symptom severity), thus leaving four items in the questionnaire.

#### Social Anxiety Disorder

In general, the experts indicated that these items accurately reflected symptoms of this anxiety disorder. Two items, “I am afraid of being bullied at school” and “I am afraid I can’t get the words out” were less satisfactory in terms of sensitivity: that is, respectively 15.9 and 22.7 % of the experts did not consider them as being indicative of social anxiety disorder. These and two other items (i.e., “I find it very scary to talk with people I don’t know” and “I am very afraid that other kids don’t like me”) also lacked specificity because they were quite frequently (i.e., between 22.7 and 54.5 %) associated with other anxiety disorders, in particular selective mutism and generalized anxiety disorder. After removing or changing items, six social anxiety disorder items were eventually included in the final scale.

#### Panic Disorder

The sensitivity of most items referring to this anxiety disorder was good. Only the items “I am afraid that other people can see when I am panicking” and “In a big store I am afraid I will panic” were quite often (i.e., 31.8 and 34.1 %) unrelated to panic disorder. These two items were associated with other anxiety problems, namely social anxiety disorder and agoraphobia respectively, and hence removed. In terms of specificity, problems were also detected with various items reflecting physical symptoms (i.e., palpitations, sweating, shaking, and dizziness) that occur during anxiety, for which various experts consistently indicated that they are relevant for *all* anxiety disorders. Two of these physical symptoms were modified and retained, and together with four other satisfactory items the final subscale comprised a total of six items.

#### Generalized Anxiety Disorder

Almost all items that intended to assess this anxiety disorder showed good sensitivity and specificity. The only exception was the item “I worry a lot about disasters (for example earthquake, flood)”, which 70.5 % of the experts associated with a specific phobia—natural environment type and hence was removed. After discarding two redundant/somewhat ambiguous items, six items were preserved for the final questionnaire.

#### Specific Phobia: Animal Type

All five items referring to this type of specific phobia displayed excellent sensitivity as well as specificity and thus were retained in the scale.

#### Specific Phobia: Natural Environment Type

Two out of four items measuring this type of specific phobia appeared to have insufficient face validity. The first item was “I am afraid of the dark”, which did not show adequate sensitivity and specificity. Quite a number of experts thought that this item was indicative of a specific phobia—situational or other type, but in essence this fear seems to have its origins in the natural environment. In spite of this problem, we decided to retain this item because it is quite common in children [32, 33]. The other item was “I am afraid of standing on a high place”, which was also quite often attributed to the situational phobia category. However, this may have been due to the rather abstract formulation of this item and therefore we changed this item in “I am afraid of heights”, thereby covering this type of fear in a more straightforward way.

#### Specific Phobia: Blood–Injection–Injury Type

Two out of three items were satisfactory in terms of sensitivity and specificity. The item that did less well in this regard was “I am afraid of getting a physical examination in the hospital”, which was not identified as belonging to this type of phobia by 18.2 % of the experts and was frequently (i.e., 29.5 %) associated with a range of other anxiety problems such as situational phobia, social anxiety, and generalized anxiety (although none of these exceeded the 10 % criterion). In order to strengthen its relation to blood–injection–injury phobia, the item was modified into “I am afraid of undergoing a small medical operation”.

#### Specific Phobia: Situational Type/Agoraphobia

Situational phobia and agoraphobia are similar in terms of clinical presentation as they show clear “overlap in feared situations” [21, p. 201], which justifies why these anxiety problems were combined in our measure. Six out of 10 items displayed moderate face validity figures, leading to a fairly low overall sensitivity of 85.9 %, while problems with specificity were noted by 30.0 % of the experts. More specifically, a substantial proportion of the experts linked the item “I am afraid to travel in an airplane” to specific phobia—other type, “I am afraid of places with a lot of people” to social anxiety disorder, “I am afraid when travelling by car” to generalized anxiety disorder (38.6 %, probably because this item was associated with worry about being involved in an accident), “I am afraid to cross a long bridge” and “I am afraid when sailing on a boat” to natural environment phobia, and “I am afraid to go outside on my own” to separation anxiety disorder. After either discarding or changing a number of these problematic items, six items were retained for the final questionnaire.

#### Specific Phobia: Other Type

By definition, this is a residual category and, as such, it was hardly surprising that its face validity was limited. “I am afraid of loud noises” was frequently identified by experts as a symptom of natural environment phobia. “I am afraid that I will choke” was quite often classified as panic disorder, and “I am afraid that I have to vomit” as panic disorder or blood–injection–injury phobia. We decided to retain these four items, although the emetophobia and choking phobia items were slightly rephrased to improve their coverage of these phobic problems [34, 35].

### Construction of the Final Version of the Questionnaire

As a result of this process, a total of 50 items was retained for the final questionnaire. We decided to create two separate parts: Part I (i.e., YAM-5-I) consisted of 28 items and was devoted to the major anxiety disorders and included separation anxiety disorder, selective mutism, social anxiety disorder, panic disorder, and generalized anxiety disorder, whereas Part II (i.e., YAM-5-II) contained 22 items and was concerned with the specific phobias including agoraphobia (mainly by virtue of the fact that this anxiety disorder was merged with situational phobia). As a response format, a four-point Likert scale was chosen, with 0 = never, 1 = sometimes, 2 = often, and 3 = always. As noted previously, besides the child (self-report) version of the YAM-5, there is also a parent version which asks the mother and/or father to rate the frequency of their offspring’s anxiety disorder and phobia symptoms from their point of view.

There are several reasons for the division between major anxiety disorders and specific phobias in the YAM-5. First of all, empirical studies investigating the structure of negative emotions in youth have indicated that anxiety and fear (phobia) symptoms are separate (yet correlated) components of negative emotions [36]. Second, this notion is also supported by a review of Sylvers et al. [37] who concluded that anxiety is more future-focused and diffuse, and characterized by hypervigilance during the approach of a potential threat. In contrast, fear is more present-focused and specific, and typified by fight–flight–freeze responses facilitating escape from threat. Third, negative cognitions seem to be more developed and elaborated in anxiety than in fear conditions [38], and this has also been extended to the development of treatment approaches, which primarily include cognitive techniques for the major anxiety disorders but mainly focus on exposure for the specific phobias and agoraphobia [39]. Fourth, previous factor analytic studies have indicated that it is almost impossible to find a satisfactory structure for comprehensive childhood anxiety measures [40] and that phobia items are particularly problematic in this psychometric conundrum. On the one hand these fears, due to their specific nature, do not necessarily form a homogeneous cluster with other fears or phobias. On the other hand these fears are often found to be associated with major anxiety disorders [41]. Finally, in research settings, the major anxiety disorders are often studied separately from the specific phobias. Thus by construing a measure consisting of two parts, it becomes possible to employ a fairly short scale for measuring either symptoms of the major anxiety disorders or the phobias.

### Second Face Validity Check by Clinicians

The two parts of the YAM-5 (i.e., YAM-5-I: major anxiety disorders and YAM-5-II: specific phobias including agoraphobia) were then given to a panel of clinical experts, comprising child psychologists and psychiatrists who were regularly confronted with children and adolescents with anxiety disorders in daily practice. The instruction for the clinical experts was to indicate for each item which anxiety disorder or phobia it most likely represented. Thus, the face validity check by the clinicians was conducted in a more stringent way than the approach used by the research experts who were allowed to indicate various anxiety disorders/phobias for each item and to comment on the quality of the items. The results of this second face validity check indicated that most items listed in Part I, the major anxiety disorders, showed satisfactory sensitivity and specificity (Table 2). The only exception was selective mutism: a substantial proportion of the clinical experts (19.0 %) had difficulty linking these items correctly to this new anxiety disorder, with most of them interpreting the symptoms as indicative of social anxiety disorder. Given the rarity of selective mutism, it is possible that some professionals lacked the knowledge to link its items correctly to the disorder.

**Table 2:** Results of the face validity check of the final 28 YAM-5-I items as performed by the clinicians (*N* = 34) as well as reliability estimates (item–total correlations and Cronbach’s alpha coefficients; left values: non-clinical adolescent sample, *N* = 132 and right values: clinically referred youths, *N* = 64) for various anxiety disorders subscales and the total scale

Anxiety disorder item (number in final scale)	Confirmation by clinicians (%) (sensitivity)	Alternative anxiety disorder (%)^a^ (specificity)	Item–total correlations and alpha subscale		Item–total correlations and alpha total scale	
*Separation anxiety disorder*						
I am afraid to go anywhere without my parents (1)	97.1	2.9	0.44	0.53	0.54	0.52
I get frightened if my parents leave the house without me (6)	100	0	0.48	0.51	0.36	0.41
I am afraid that my parents will leave and never come back (10)	100	0	0.72	0.83	0.60	0.65
I am afraid that something bad will happen, so I’ll never see my parents again (15)	100	0	0.64	0.62	0.57	0.61
I have very scary dreams that I lose my parents (19)	94.1	5.9	0.51	0.68	0.47	0.55
I don’t feel well when I have to go somewhere without my parents (24)	97.1	2.9	0.63	0.54	0.55	0.60
	**98.0**	**2.0**	**0.80**	**0.84**		
*Selective mutism*						
At school I don’t speak to the teacher at all (2)	91.2	8.8	0.52	0.15	0.47	0.11
If I meet a new person, I don’t speak at all (11)	68.0	32.0 (SOC)	0.51	0.46	0.49	0.58
At school I don’t speak at all to the kids in my class (20)	82.4	17.6 (SOC)	0.38	0.35	0.32	0.37
I don’t speak at all when there is a new visitor at our home (25)	82.4	17.6 (SOC)	0.53	0.42	0.39	0.65
	**81.0**	**19.0**	**0.65**	**0.55**		
*Social anxiety disorder*						
I find it scary to meet new people (3)	100	0	0.67	0.64	0.72	0.45
I find it scary to eat or drink if other people are looking at me (7)	100	0	0.61	0.47	0.67	0.55
I am afraid that others will see that I blush (12)	100	0	0.54	0.44	0.51	0.48
I am afraid I’ll do something embarrassing (16)	100	0	0.63	0.62	0.64	0.59
I am very afraid that other kids don’t like me (23)	100	0	0.57	0.53	0.62	0.51
I am afraid that I might do or say something stupid in front of others (28)	100	0	0.46	0.71	0.52	0.66
	**100**	**0**	**0.81**	**0.81**		
*Panic disorder*						
I panic for no reason (4)	100	0	0.70	0.56	0.73	0.56
I suffer from anxiety or panic attacks (8)	97.1	2.9	0.70	0.62	0.66	0.46
All of a sudden I become so scared that my heart starts to beat very quickly (13)	97.1	2.9	0.33	0.60	0.40	0.58
When I panic, I am afraid that I could die (17)	100	0	0.30	0.63	0.35	0.50
I have severe anxiety attacks during which I tremble all over my body (21)	94.1	5.9	0.59	0.43	0.54	0.41
I am afraid of having a new anxiety or panic attack (26)	97.1	2.9	0.59	0.70	0.59	0.61
	**97.9**	**2.1**	**0.76**	**0.82**		
*Generalized anxiety disorder*						
I worry about a lot of things (5)	100	0	0.76	0.75	0.67	0.64
I think a lot about what can go wrong (9)	97.1	2.9	0.63	0.55	0.68	0.48
I find it hard to stop worrying (14)	100	0	0.80	0.66	0.68	0.49
I worry a lot about not doing well at school (18)	91.2	8.8	0.65	0.44	0.55	0.30
I worry a lot about all the bad things than happen in the world (22)	94.1	5.9	0.30	0.45	0.41	0.40
I don’t feel well because I worry so much (27)	100	0	0.71	0.73	0.67	0.60
	**97.1**	**2.9**	**0.85**	**0.83**		
					**0.93**	**0.92**

YAM-5-I = Youth Anxiety Measure for DSM-5, Section I. Major anxiety disorders

*SOC* Social anxiety disorder

^a^Alternative anxiety disorder(s) is (are) only specified if indicated by more than 10 % of the experts. Clinicians were only allowed to make one choice per item, so sensitivity and specificity percentages add up to exactly 100 %. Average percentages per anxiety disorder and Cronbach’s alpha coefficients are printed in bold

The face validity check performed by the clinicians of the items listed in Part II, the phobias, indicated that three out of five phobia scales showed sub-optimal sensitivity/specificity (see Table 3). First, for natural environment phobias, the problems were caused by the items “I am afraid of the dark”, “I am afraid of heights”, and “I am afraid to swim in deep water”, which were quite often misjudged as belonging to the situational phobias. Second, of the situational phobia/agoraphobia category, the item “I am afraid when travelling by bus or train” was frequently labelled as a specific phobia—other type. Third, in the specific phobia—other type category, the clinical experts experienced some ambiguity with regard to all items. Most problematic in this regard was the item “I am afraid of loud noises”, which was often judged as belonging to the natural environment or situational phobias.

**Table 3:** Results of the face validity check of the final 22 YAM-5-II items as performed by the clinicians (*N* = 34) as well as reliability estimates (item–total correlations and Cronbach’s alpha coefficients; left values: non-clinical adolescent sample, *N* = 132, and right values: clinically referred youths, *N* = 64) for various phobias subscales and the total scale

Phobia item (number in final scale)	Confirmation by clinicians (%) (sensitivity)	Alternative phobia (%)^a^ (specificity)	Item–total correlations and alpha subscale		Item–total correlations and alpha total scale	
*Specific phobia—animal type*						
I am afraid of wasps (1)	100	0	0.53	0.42	0.61	0.51
I am afraid of dogs (3)	100	0	0.23	0.21	0.35	0.15
I am afraid of spiders (9)	100	0	0.44	0.39	0.48	0.56
I am afraid of snakes (13)	100	0	0.59	0.42	0.50	0.38
I am afraid of cats (18)	100	0	0.28	0.38	0.15	0.24
	**100**	**0**	**0.66**	**0.59**		
*Specific phobia—natural environment type*						
I am afraid of the dark (4)	64.7	35.3 (SITAGO)	0.51	0.37	0.67	0.49
I am afraid of heights (6)	76.5	23.5 (SITAGO)	0.17	0.38	0.25	0.41
I am afraid of thunderstorms (10)	97.1	2.9	0.21	0.48	0.47	0.49
I am afraid to swim in deep water (12)	82.4	17.6 (SITAGO)	0.21	0.33	0.30	0.55
	**80.2**	**19.8**	**0.47**	**0.61**		
*Specific phobia—blood–injection–injury type*						
I am afraid of getting an injection (11)	100	0	0.46	0.35	0.42	0.42
I am afraid of undergoing a small medical operation (15)	94.1	5.9	0.49	0.45	0.65	0.64
I am afraid of blood (19)	100	0	0.47	0.49	0.54	0.54
	**98.0**	**2.0**	**0.65**	**0.62**		
*Specific phobia—situational type/Agoraphobia*						
I am afraid to travel in an airplane (5)	91.2	8.8	0.51	0.42	0.52	0.58
I am afraid when crossing a large town square (7)	88.3	11.7	0.58	0.23	0.54	0.13
I am afraid of being in crowded places with lots of people (16)	100	0	0.51	0.68	0.48	0.58
I am afraid when travelling by bus or train (17)	85.3	14.7 (OTH)	0.51	0.55	0.41	0.50
I am afraid to go in an elevator (21)	88.3	11.7	0.37	0.42	0.41	0.50
I am afraid to go through a long tunnel (22)	91.2	8.8	0.52	0.49	0.51	0.43
	**90.8**	**9.2**	**0.74**	**0.67**		
*Specific phobia—other type*						
I am afraid of loud noises (2)	52.9	47.1 (NATENV, SITAGO)	0.19	0.30	0.39	0.44
I am afraid of people who are dressed up in costumes (8)	88.3	11.7 (SITAGO)	0.29	0.17	0.46	0.33
I am afraid that I will feel sick and have to vomit (14)	88.3	11.7	0.34	0.17	0.40	0.40
I am afraid choking when I eat or drink (20)	85.3	14.7 (SITAGO)	0.30	0.29	0.40	0.51
	**78.7**	**21.3**	**0.47**	**0.41**		
					**0.86**	**0.86**

YAM-5-II = Youth Anxiety Measure for DSM-5, Section II. Phobias

*SITAGO* specific phobia—situational type/agoraphobia, *OTH* specific phobia—other type, *NATENV* specific phobia—natural environment type

^a^Alternative phobia(s) is (are) only specified if indicated by more than 10 % of the experts. Clinicians were only allowed to make one choice per item, so sensitivity and specificity percentages add up to exactly 100 %. Average percentages per disorder and Cronbach’s alpha coefficients are printed in bold

### Suitability

In both samples, youths received explicit instructions to call upon the research assistant (non-clinical sample) or the test diagnostician (clinical sample) in case they had any questions about the YAM-5 items. In the non-clinical sample, youths appeared to experience no difficulties while completing the new questionnaire: there were few questions about items and almost no missing values. The clinical sample also included younger children and here there were slightly more questions, but on the whole children completed the scale without obvious problems. Only children with a specific learning disorder (with impairment in reading) needed assistance to complete the measure: in these cases, items were read aloud by the diagnostician while the child read along and rated the items. While applying this procedure, children appeared to show good understanding of the items. These observations suggest that the scale is acceptable and suitable for measuring anxiety symptoms in youths aged 8 years or older.

### Reliability

The reliability of both the YAM-5-I and YAM-5-II was investigated by computing item–total correlations and Cronbach’s alphas. As shown in Table 2, the Cronbach’s alpha for the total scale of YAM-5-I (major anxiety disorders) was excellent (α = 0.93 in the non-clinical sample and α = 0.92 in the clinical sample), with item–total correlations mostly being in the acceptable range (*r*’s between 0.32 and 0.73 in the non-clinical and between 0.11 and 0.66 in the clinical sample). For the separate subscales, internal consistency coefficients were quite good and comparable for the nonclinical and clinical sample: that is, most Cronbach’s alphas were between 0.76 and 0.85, and item–total correlations were substantial. The only exception was the selective mutism subscale, which displayed alphas of 0.65 (item–total *r*’s between 0.38 and 0.53) in the non-clinical sample and 0.55 (item–total *r*’s between 0.15 and 0.46) in the clinical sample.

The Cronbach’s alpha for the total scale of YAM-5-II (phobias) was also good (α = 0.86 in both the non-clinical and clinical sample), with item–total correlations ranging between 0.13 and 0.67 (Table 3). For various subscales, alpha values were in the moderate to sufficient range (i.e., between 0.60 and 0.75), but for animal phobia (clinical sample: α = 0.59), environmental phobia (non-clinical sample: α = 0.47) and other phobia (non-clinical sample: α = 0.47, clinical sample: α = 0.41) this type of reliability was insufficient. In general, item–total correlations for the YAM-5-II subscales varied between 0.17 and 0.59 in the non-clinical and between 0.17 and 0.68 in the clinical sample.

### Parent–Child Agreement

Table 4 presents the psychometric findings with regard to the parent version of the YAM-5, which was only completed for the clinically referred children and adolescents. First, it was found that the reliability of the YAM-5-I (major anxiety disorders) total scale was excellent, with a Cronbach’s alpha of 0.91 and item–total correlations varying between 0.10 and 0.79. The internal consistency coefficients for the subscales of the YAM-5-I parent version were also good, with again selective mutism being the exception to this rule (α = 0.65). The reliability of the YAM-5-II (phobias) total scale was good, with an alpha of 0.77 and item–total correlations between 0.10 and 0.58. The internal consistency coefficients for the subscales appeared to be rather poor. Only the Cronbach’s alpha of the blood–injection–injury phobia subscale was satisfactory (α = 0.86), but all other phobia subscales produced reliability coefficients lower than 0.60.

**Table 4:** Psychometric findings regarding the parent version of the YAM-5 that was administered in the clinical sample (*N* = 63^a^): Reliability coefficients (range item–total correlations) and parent–child agreement

	Parent version	Cronbach’s α (item–total *r*’s)	Child version	*r* (parent–child)^‡^	*t* value
*YAM-5-I anxiety disorders*	15.38 (10.20)	0.91 (0.16–0.79)	17.21 (12.40)	0.69	1.59
Separation anxiety disorder	2.35 (2.70)	0.84 (0.44–0.71)	2.44 (3.06)	0.73	0.35
Selective mutism	2.04 (1.97)	0.64 (0.16–0.67)	2.22 (2.23)	0.42	0.61
Social anxiety disorder	4.03 (3.31)	0.85 (0.55–0.74)	4.71 (3.70)	0.67	1.89
Panic disorder	1.51 (2.26)	0.86 (0.51–0.76)	2.49 (3.14)	0.65	3.26**
Generalized anxiety disorder	5.44 (3.82)	0.87 (0.50–0.84)	5.33 (3.90)	0.68	−0.28
*YAM-5-II phobias*	10.02 (6.55)	0.77 (0.10–0.58)	11.71 (8.74)	0.70	2.16*
Animal type	4.02 (2.62)	0.47 (0.16–0.36)	4.00 (2.89)	0.61	−0.05
Natural environment type	1.86 (1.94)	0.53 (0.22–0.45)	2.44 (2.35)	0.55	2.25*
Blood–injection–injury type	2.13 (2.46)	0.86 (0.64–0.85)	2.05 (1.95)	0.64	−0.33
Situational type/agoraphobia	0.78 (1.16)	0.35 (0.12–0.26)	1.51 (2.15)	0.62	3.42**
Other type	1.24 (1.46)	0.41 (0.13–0.41)	1.71 (1.81)	0.58	2.47*

YAM-5 = Youth Anxiety Measure for DSM-5

^a^For one child, YAM-5 parent version data were not available

^‡^All parent–child correlations were significant at *p* ≤ 0.001

* *p* < 0.05; ** *p* < 0.01

The parent–child agreement appeared to be quite good, with correlations of 0.69 for YAM-5-I (major anxiety disorders) and 0.70 for YAM-5-II (phobias) total scales and between 0.42 (selective mutism) and 0.73 for separation anxiety disorder for various subscales. Note further that symptoms of panic disorder, natural environment phobia, situational/agoraphobia, other phobia, and total phobia symptoms were rated as more frequent and intense by children themselves than by parents.

### Validity

The correlations between the YAM-5-I and YAM-5-II total scores and the other measures that were taken in the clinical sample are shown in Table 5. As can be seen, the YAM-5 scales were significantly and positively correlated with anxiety symptoms as reported during the Junior SCID interview (*r*’s between 0.36 and 0.64), which of course provides evidence for the concurrent validity of the measure. There was also support for the convergent and divergent validity of the YAM-5. That is, a number of significant positive correlations were found with the Achenbach scales measuring internalizing problems, while no significant links were noted between the YAM-5 scores and externalizing. The strongest links were observed between the child version of the YAM-5-I (major anxiety disorders) and YSR internalizing (*r* = 0.52), and between the parent version of the YAM-5-I (major anxiety disorders) and CBCL internalizing (*r* = 0.54).

**Table 5:** Findings on the concurrent (i.e., correlations with interview-assessed anxiety symptoms), convergent (i.e., correlations with internalizing), and divergent (i.e., correlations with externalizing problems) validity of the YAM-5 as obtained in the sample of clinically referred youths

	*n*	YAM-5 Child version		YAM-5 Parent version	
		I Anxiety disorders	II Phobias	I Anxiety disorders	II Phobias
SCID junior—anxiety symptoms	62	0.53**	0.39*	0.64**	0.36*
CBCL internalizing	55	0.31*	0.23	0.54**	0.32*
CBCL externalizing	55	−0.03	0.13	0.08	0.23
YSR internalizing	33	0.52*	0.12	0.28	0.04
YSR externalizing	33	−0.06	−0.18	−0.09	−0.09

*SCID Junior* Junior version of the Structured Clinical Interview for DSM-5, *YAM-5* Youth Anxiety Measure for DSM-5, *CBCL* Child Behavior Checklist, *YSR* Youth Self-Report

* *p* < 0.05; ** *p* < 0.01

**Fig. 1:**
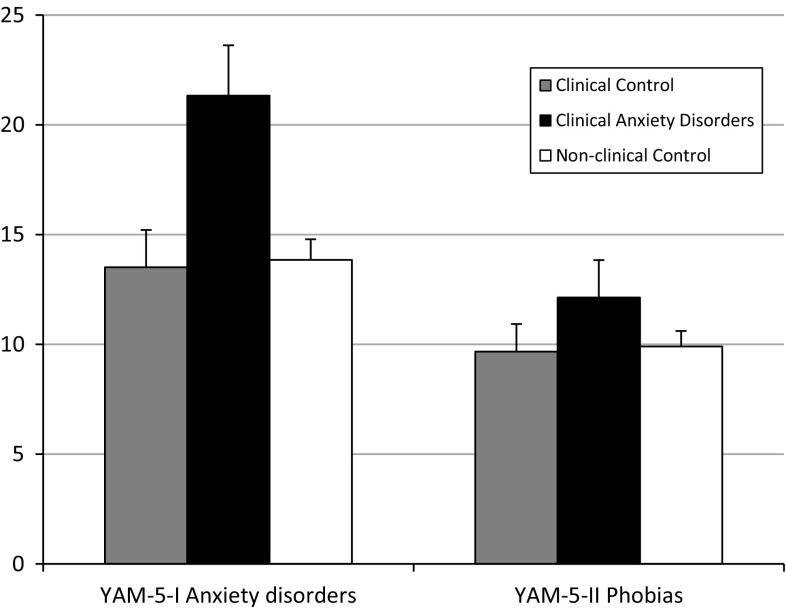
Mean YAM-5 scores (corrected for age and gender) and SE of clinically referred youth with and without anxiety disorders (*n*’s being 21 and 43) and non-clinical youth (*N* = 132). *Note*. YAM-5 = Youth Anxiety Measure for DSM-5. Only on YAM-5-I Anxiety disorders, the clinical anxiety disorders group displayed significantly higher scores than the other two groups (*p* < 0.05)

In order to get a first impression of the discriminant validity of new scale, analyses of variance were conducted to compare the YAM-5-I and YAM-5-II scores of the non-clinical adolescents, the clinically referred youths with anxiety disorders, and the clinically referred youths with other problems. As these three groups were not comparable in terms of age and gender, these variables were included in the analyses as covariates (i.e., ANCOVAs). The results of these analyses indicate that there was no significant difference among the three groups on the YAM-5-II (phobias) [*F*(1,191) < 1]. However, as shown in Fig. 1, the three groups did differ in terms of YAM-5-I (major anxiety disorders) scores [*F*(1,191) = 4.95, *p* < 0.01]. Post-hoc tests indicated that it was the group of clinically referred youths with anxiety disorders that scored significantly higher on this scale than the other two groups (both *p*’s < 0.05).

## Discussion

This article describes the development of the YAM-5, a new scale for measuring anxiety disorder symptoms in children and adolescents according to the contemporary psychiatric classification system (DSM-5). Two international panels of experts, one consisting of childhood anxiety researchers and one of clinicians working with this type of psychopathology in daily practice, were used to develop, improve, and confirm the validity of items that were intended to measure the symptoms of the separate anxiety disorders as defined in the current edition of the DSM (i.e., DSM-5). A final questionnaire consisting of 50 items was constructed that is composed of two parts. The first part (i.e., YAM-5-I) measures symptoms associated with the major anxiety disorders and contains items referring to separation anxiety disorder, selective mutism, social anxiety disorder, panic disorder, and generalized anxiety disorder. The second part (i.e., YAM-5-II) assesses symptoms associated with the specific phobias and also includes agoraphobia, which in terms of item content is difficult to discern from situational phobias [21]. With both parts combined, the YAM-5 assesses symptoms of the full spectrum of anxiety disorders that may occur in children and adolescents.

The process of constructing this questionnaire underscores the difficulties in classifying anxiety disorders in terms of fear/anxiety content alone (as expressed by core symptoms), which is the key principle of the DSM. The main issue here is differential diagnosis: it has been noted that even when only considering anxiety problems, it may still be quite hard to identify the correct anxiety disorder in relation to a given symptom [42]. Illustrative in this regard are the lack of a clear distinction between situational phobia and agoraphobia, and the problems experienced by our experts in discerning between selective mutism and social anxiety disorder, and among the different types of phobias. Another source of confusion originates from the fact that the central feature of one anxiety disorder can overlap and be present in other anxiety disorders. Good examples are panic attacks, which are typical of panic disorder but also frequently occur in other anxiety disorders [43], and furthermore worry, which is most characteristic of generalized anxiety disorder but is also often present in other anxiety problems [44]. We addressed the attribution of symptoms to incorrect anxiety disorders by describing the symptoms more specifically and deleting items that were ambiguous and could not be reformulated in a more specific way. Even though effort was made to design items for the YAM-5 that are as specific as possible to each anxiety disorder, it is clear that the problem of differential diagnosis cannot be completely resolved in this type of questionnaire. Therefore, we would like to emphasize here that although scales such as the YAM-5 can be very useful as an index of symptom frequency/intensity in various anxiety domains, they can never replace a standardized diagnostic interview in order to establish the presence of anxiety disorders in a child or adolescent [7].

As noted earlier, there were a number of reasons for our decision to split our anxiety measure in two parts, one part covering the major anxiety disorders and another part encompassing the specific phobias/agoraphobia. A critical point can be raised regarding the fact that in the YAM-5 agoraphobia is grouped with the specific phobias. We have already clarified that the main reason for this was that on an item (symptom) level, it is not possible to differentiate agoraphobia from a situational specific phobia. In spite of this, there might also be good arguments for placing agoraphobia with the major anxiety disorders. First, agoraphobia frequently co-occurs with panic disorder in adults [45], and this appears also true in children and adolescents [46]. Second, there are clear indications that catastrophic cognitions are quite elaborated in agoraphobia [47], and this feature shows more resemblance to the major anxiety disorders than to the specific phobias. Third and finally, when looking at clinical presentation, agoraphobia is more severe and impairing than specific phobias [48], and, as such, more affiliated with the major anxiety disorders. For those researchers and clinicians who prefer to assess agoraphobic symptoms alongside the major anxiety disorders (but do not want to assess other types of specific phobias), we created a YAM-5 Part I + version which includes both the major anxiety disorders and agoraphobia.

The children and adolescents who tested the YAM-5 did not report noteworthy difficulties with completing the scale. Internal consistency coefficients of the total anxiety disorders and phobias scales were good to excellent, whereas the reliability estimates for most subscales were in the acceptable to good range. There were a number of exceptions to this rule: for example, the internal consistency of the selective mutism scale was insufficient (clinical sample) and the same was also true for a number of phobia scales (both samples). In the case of selective mutism, the low alpha value may be due to the fact that this subscale taps a low-frequent anxiety problem by means of a limited set of items. Further, for the ‘other phobia’ subtype, low consistency could have been anticipated as this is by definition a residual category. However, for animal phobia (clinical sample) and natural environment phobia (non-clinical sample, the rather low reliability coefficients were less expected. Meanwhile, there is also research demonstrating that animal phobias are quite heterogeneous and consist of various dimensions [49], whereas natural environment phobias have not always emerged as a separate category but rather tend to blend with situational phobias [50]. It is good to keep in mind that phobias are by definition specific, and it may not be feasible to expect them to actually cluster with other phobias into the categories as described in the DSM.

Data on the parent version of the YAM-5 were obtained in the clinical sample and yielded a number of interesting findings. First, reliability coefficients generally showed a similar pattern as those found for the child version. There was one additional subscale that produced an extremely low Cronbach’s alpha, namely situational/agoraphobia, but note that symptoms of this anxiety problem were hardly endorsed by the parents, which may have caused a restriction-of-range problem. Second, the parent–child agreement of the YAM-5 was good, with mean *r*’s of 0.64 for YAM-5-I (major anxiety disorders) and 0.62 for YAM-5-II (phobias). These cross-informant figures compare favorably with those generally obtained in research on internalizing symptoms (with an overall mean r of 0.25 [51]), which can be explained by the fact that this was a clinical sample in which parents had relatively good awareness of their offspring’s anxiety problems. Third and finally, on some YAM-5 (sub)scales parents displayed significantly lower sores than children. This result is in agreement with what has been reported in the literature, namely that anxiety is an internalizing problem of which not all symptoms are overt and visible, even for children’s daily caregivers [52].

First evidence was also found for the validity of the new scale. To begin with, YAM-5 scores correlated positively and significantly with the number of anxiety symptoms as reported by youths and parents during a structured clinical interview, which provides support for the concurrent validity. Further, indications were found for the convergent and divergent validity: that is, significant positive associations were noted with the internalizing scales of the Achenbach questionnaire, whereas no substantial links were observed with the externalizing scales of this measure. Finally, clinically referred youths with anxiety disorders scored higher on the YAM-5-I scale (major anxiety disorders) as compared to clinically referred youths with other problems and non-clinical controls, suggesting that the measure has discriminant validity. The test of the validity of the YAM-5 was not optimal: the sample size of clinically referred children and adolescents was rather small and there were quite a number of youths with anxiety disorder not otherwise specified. Future investigations should be conducted in larger clinical samples with more variation in anxiety problems so that the discriminant validity can also be explored at a subscale level. In addition, other aspects of reliability (i.e., test–retest reliability) and validity (e.g., factor structure, treatment sensitivity) of the new measure need to be examined.

It is increasingly acknowledged that anxiety problems are situated on a continuum with low fear and anxiety at one end of a dimension, and high fear and anxiety or even phobias and anxiety disorders (whereby there is significant interference with daily functioning) at the other end [53]. As the YAM-5 is based on the *content* of fear and anxiety, which is the defining principle for the anxiety disorders as described in the DSM-5, one might have the impression that the scale is more or less a categorical measure. This is not intended to be the case: the scale quantifies the frequency/intensity of fear and anxiety, and, as such, adopts a dimensional approach, while assessing fear and anxiety symptoms that reflect the current diagnostic classifications of anxiety disorders thereby bridging the gap for clinicians and researchers who rely on the categorical approach.

## Summary

The present article describes the development of the YAM-5, a new questionnaire for assessing anxiety disorder symptoms in children and adolescents in terms of the contemporary classification system, the DSM-5. International panels of childhood anxiety researchers and clinicians were consulted to construct a scale consisting of two parts: Part I (i.e., YAM-5-I) consists of 28 items and measures the major anxiety disorders including separation anxiety disorder, selective mutism, social anxiety disorder, panic disorder, and generalized anxiety disorder, whereas Part II (i.e., YAM-5-II) contains 22 items and is concerned with the specific phobias including agoraphobia. In general, the face validity of the new scale proved to be acceptable: most of its items were successfully linked to the intended anxiety disorders and phobias. A first test of the YAM-5 in two samples of non-clinical adolescents and clinically referred youths indicated that the measure was easy to complete. Further, support was found for the internal consistency reliability of the new measure as well as its parent–child agreement and concurrent, convergent, divergent, and discriminant validity. In summary, the YAM-5 represents a potentially important addition to the assessment toolbox of clinicians and researchers who want to evaluate the level of anxiety disorder symptoms in children and adolescents. This DSM-based measure quantifies symptoms in a relatively brief, cost-effective, and reliable manner, and is particularly useful in situations where a diagnostic interview is not feasible. However, more studies on the psychometric qualities of the scale and collection of normative data in both non-clinical and clinical populations of children and adolescents are urgently needed.
